# Serum CD5L Responds Positively to Selenium and Coenzyme Q_10_ Supplementation with Relation to Thyroid Hormones, Mortality, and Health-Related Quality-of-Life—A Sub-Analysis of a Double-Blind Randomised Placebo-Controlled Trial in Elderly Low in Selenium

**DOI:** 10.3390/antiox14030366

**Published:** 2025-03-20

**Authors:** Urban Alehagen, Jan O. Aaseth, Trine B. Opstad, Anders Larsson, Sabrina Asaad, Lutz Schomburg, Jan Alexander

**Affiliations:** 1 Division of Cardiovascular Medicine, Department of Medical and Health Sciences, Linköping University, 581 85 Linköping, Sweden; 2 Department of Research, Innlandet Hospital Trust, 2380 Brumunddal, Norway; jaol-aas@online.no; 3 Faculty of Health and Social Sciences, University of Inland Norway, 2605 Lillehammer, Norway; 4 Oslo Center for Clinical Heart Research—Laboratory, Department of Cardiology, Oslo University Hospital Ullevål, 0450 Oslo, Norway; t.b.opstad@medisin.uio.no; 5 Faculty of Medicine, University of Oslo, 0372 Oslo, Norway; 6 Department of Medical Sciences, Uppsala University, 751 85 Uppsala, Sweden; anders.larsson@akademiska.se; 7 Institut für Experimentelle Endokrinologie, Charité-Universitätsmedizin Berlin, 10115 Berlin, Germany; sabrina.asaad@charite.de (S.A.); lutz.schomburg@charite.de (L.S.); 8 Norwegian Institute of Public Health, 0213 Oslo, Norway; jan.alexander@fhi.no

**Keywords:** CD5L, elderly, selenium, supplementation, thyroid hormones, quality-of-life

## Abstract

The Cluster of Differentiation 5-like protein (CD5L) is produced by tissue-resident macrophages. It is an innate immune mediator protein with a multitude of functions, such as binding of invading microorganisms and oxidised LDL, and it is associated with clinical conditions, i.e., atherosclerosis and inflammation. The circulating CD5L level has been reported to correlate to selenium status and thyroid hormone activity. In order to test this hypothesis, we analysed CD5L in serum samples from a randomized controlled trial (RCT) with selenium and coenzyme Q_10_ supplementation and examined associations between CD5L and thyroid hormones, health-related quality-of-life (Hr-QoL), and mortality in an elderly population low in selenium. Circulating levels of CD5L and thyroid hormones were determined in 359 elderly community-living individuals enrolled in an RCT at inclusion and after 48 months of supplementation (179 received selenium and coenzyme Q_10_, and 180 placebo). Hr-QoL was recorded at both time-points using Short Form 36. Pre-intervention plasma selenium was low, mean 67 µg/L. CD5L correlated positively to free tri-iodothyronine (fT3) and showed an inverse relation with thyroid stimulating hormone (TSH). Low CD5L concentrations at inclusion in the placebo group were associated with increased cardiovascular mortality during 10 years of follow-up, and impaired Hr-QoL at 48 months. Selenium and coenzyme Q_10_ supplementation significantly increased CD5L and fT3 levels, in association with a better health outcome. The data indicate that circulating CD5L positively responds to selenium and coenzyme Q_10_ supplementation, correlates with thyroid hormone status, and associates with positive health indices. The observed effect may be due to increased selenium-dependent deiodinase isozyme expression that converts thyroxine (T4) to T3 locally and supports thyroid hormone activities. Whether the observed associations with Hr-QoL and cardiovascular mortality are a direct effect of circulating CD5L or local thyroid hormone activity is unclear and should be further investigated.

## 1. Introduction

Selenium is an essential trace element that occurs as selenocysteine in selenoproteins, which are important for protection against oxidative stress, inflammation, and thyroid metabolism [1]. The endogenous production of coenzyme Q_10_ (CoQ_10_), which is reduced in the elderly [2], is a powerful lipid-soluble antioxidant that works in concert with selenium [3,4,5]. In a recent study, we found that low selenium in an elderly population was associated with low activity of selenium-dependent deiodinases and low serum free tri-iodothyronine (fT3). Upon supplementation with selenium and CoQ_10_, deiodinase activity appeared to increase along with beneficial clinical effects [6]. A close relationship between selenium and the thyroid is already convincingly shown in the literature [7].

Circulating cell surface Cluster of Differentiation 5-like protein (CD5L) is an innate immune effector protein of 347 amino acids mainly secreted from tissue-resident macrophages in the liver (Kupffer cells). In the literature, CD5L is also described as an “Apoptosis Inhibitor of Macrophage” (AIM). CD5L inhibits macrophage apoptosis and therefore supports their survival [8,9]. One of the mechanisms in which CD5L is involved in immunity is its role in binding to invading bacteria and to fungi during infections [10,11], preparing for protective phagocytosis. CD5L is linked to lipid metabolism as its expression in macrophages is regulated by the LXR receptor and its main ligand, 25-hydroxy cholesterol, with its partner RXR [12]. CD5L may also show a pro-atherosclerotic effect through binding to oxidised LDL, facilitating its uptake into foam macrophages [13]. There are some clinical reports that state the usefulness of CD5L as a biomarker. Peters et al. showed that low levels of circulating CD5L successfully identify those with a rapidly declining renal function [14]. Furthermore, an increased concentration of CD5L seems to be a valuable biomarker for early identification of graft rejection after heart transplantation [15]. CD5L also initiates the activation of inflammation through several cytokines and increases survival of pro-inflammatory synovial cells in patients with rheumatoid arthritis [16].

Yet another interesting aspect of CD5L was recently published by Nock et al., who reported that circulating CD5L is regulated by thyroid hormones [17]. They showed a significant inverse correlation between CD5L and TSH and a positive correlation between CD5L and fT3 in patients with hypo- and hyperthyroidism, respectively. It appeared that the CD5L synthesis by macrophages was not directly regulated by T3 but occurred indirectly via secretion of a yet unknown signalling factor from T3-stimulated hepatocytes. These authors further suggested that CD5L, as it depends on local thyroid hormone control, might serve as a biomarker of peripheral thyroid hormone activity beyond hypophyseal thyrotropin (TSH) in the feedback axis. This notion was recently confirmed in pregnancy, where serum CD5L correlated positively with various parameters of thyroid hormone status as well as selenium [18].

Therefore, we hypothesize that CD5L will have a relationship with selenium and that supplementation with selenium in a population low in selenium will increase also the concentration of CD5L.

The aim of the present study was to analyse a possible relationship between thyroid hormone activity and CD5L concentrations in an elderly population low in selenium, both in an observational setting and following a randomised intervention with selenium and CoQ_10_, and to evaluate any associations of CD5L with established endpoints of thyroid hormone action, including health-related quality-of-life (Hr-QoL), and cardiovascular and all-cause mortality.

## 2. Methods

### 2.1. Subjects

**Figure 1:**
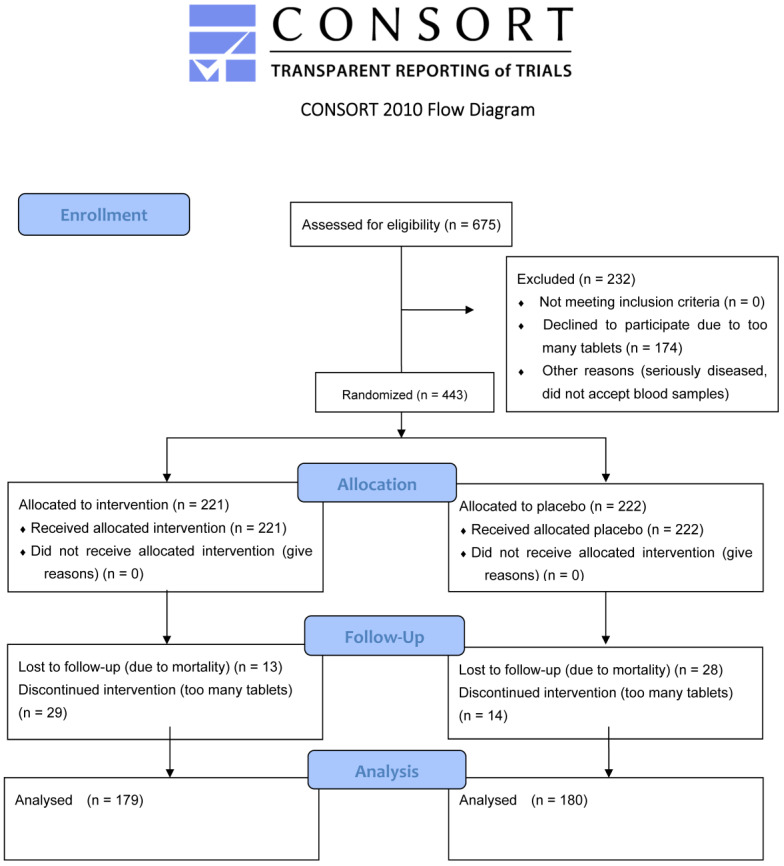
A flowchart of the total follow-up period.

All individuals aged 69–88 years living in a rural municipality in southeastern Sweden were invited to join the main epidemiological study. Of the 1320 residents, 876 agreed to participate. In 2003, 675 surviving participants from the main study were invited to take part in an intervention project involving selenium and CoQ_10_ supplementation. Some individuals declined participation due to the long distance to the health centre, refusal to undergo blood sampling, or unwillingness to take the supplements. As a result, 443 individuals aged 70–88 years agreed to participate in the intervention project. The study involved a four-year supplementation with selenium and CoQ_10_, or a placebo, with blood samples collected every six months [19] (Figure 1).

At the start of the intervention, participants had a deficient selenium status, with a mean serum selenium concentration of 67 ± 16.8 µg/L, which corresponds to an estimated daily intake of 35 µg/day—well below the adequate selenium level of ≥100 µg/L [20]. Participants received 200 mg/day of CoQ_10_ (Bio-Quinon 100 mg B.I.D, Pharma Nord, Vejle, Denmark) and 200 µg/day of organic selenium yeast (SelenoPrecise 100 µg B.I.D, Pharma Nord, Vejle, Denmark) (n = 221) or a matching placebo (n = 222) for 48 months. The supplementation was taken alongside any regular medications. Compliance was measured by counting returned, unused study medications (both active and placebo). All participants were examined by one of three experienced cardiologists. A clinical history was recorded at baseline, and clinical examinations, including blood pressure, New York Heart Association functional class (NYHA class), electrocardiograms (ECGs), and Doppler echocardiography, were performed at both baseline and after the study period. Echocardiograms were conducted with participants in the left lateral position. Ejection fraction (EF) was categorized into four classes with interclass limits set at 30%, 40%, and 50% [21,22]. Normal systolic function was defined as EF ≥ 50%, while severely impaired systolic function was defined as EF < 30%. Only systolic function was assessed. The inclusion period began in January 2003 and concluded in February 2010. The first participant was included in January 2003, and the last participant concluded the study in February 2010.

For this sub-analysis, only participants who provided blood samples throughout the entire intervention period and survived the full duration of the study were included. The final study population consisted of 359 individuals, with 179 receiving active supplementation (selenium and CoQ_10_) and 180 receiving a placebo (Table 1). Participants with known or suspected thyroid disease, or those on medication for thyroid conditions, were excluded from this sub-analysis.

### 2.2. Ethical Approval

The study received approval from the Regional Ethical Committee (Forskningsetikkommitten, Hälsouniversitetet, SE-581 85 Linköping, Sweden; No. D03-176) and adheres to the ethical standards outlined in the 1975 Declaration of Helsinki. The Medical Product Agency did not review the study protocol, as the study was not classified as a clinical trial for a specific disease medication but rather as a study on commercially available food supplements. This study was registered with ClinicalTrials.gov under the identifier NCT01443780. Since registration was not required when the study commenced, it was registered retrospectively. Written informed consent was obtained from all participants.

### 2.3. Blood Sampling

All blood samples in this sub-study were collected under fasting condition at inclusion and after 48 months and drawn while the participants were resting in a supine position.

Whole-blood vials for serum preparation were centrifuged at 2500× *g* for 10 min and kept frozen at −70 °C until the determination of CD5L and other biochemical markers (CRP, P-selectin [21], Galectin-3 [22], and intracellular adhesion molecule I (ICAM-1) [23]). Pre-chilled ethylenediaminetetraacetic acid (EDTA) vials were centrifuged at 3000× *g*, +4 °C, and EDTA plasma was frozen at −70 °C for the measurement of selenium concentration.

### 2.4. Determination of Selenium

Selenium analyses were conducted using ICP-MS methodology on an Agilent 700 platform at the Kompetenzzentrum für komplementärmedizinische Diagnostik, a branch of synlab MVZ Leinfelden GmbH (Leinfelden-Echterdingen, Germany) [24]. The accuracy of the measurements was verified by analysing two external reference materials with certified concentrations of 63 µg/L and 103 µg/L, provided by the Society for Advancement of Quality Assurance in Medical Laboratories (INSTAND e.V., Düsseldorf, Germany). The results were within 90–110% of the certified values. The laboratory consistently passed round-robin tests conducted by INSTAND e.V. The precision of the method, assessed through repeated analyses of the same serum samples, showed an average inter-assay coefficient of variation of 5.7%.

### 2.5. Determination of CD5L

The serum concentration of CD5L was assessed with a recently developed quantitative sandwich assay (ELISA) using a pair of CD5L-specific monoclonal antibodies. Assay development, specificity, linearity, precision, and matrix characteristics have been determined and are published elsewhere [18]. Briefly, serum samples were diluted and applied to pre-coated magnetic beads, the acridinium-labelled second monoclonal antibody was applied, and the components were incubated for 7.5 min. Then, the complexes were washed, and signal detection was initiated by adding alkaline hydrogen peroxide to initiate bioluminescence. The incubation and wash steps, along with signal detection and quantification, were performed using an IDS-iSYS multi-discipline automated system (ids immunodiagnostic systems, Boldon, UK). A human serum pool was used as positive control, and a commercial CD5L preparation was used for calibration (catalog #2797-CL, Bio-Techne, Minneapolis, MN, USA). Intra-assay coefficient of variation during the measurements was below 15%. All analyses were conducted by scientists blinded to the clinical information.

### 2.6. Determination of the Thyroid Hormones

Free T3 (kit: T3F31-K01), free T4 (kit: T4F31-K01), reverse T3 (kit: RT331-K01), and TSH (kit: THH31-K01) were analysed by use of commercial ELISA kits (Eagle Biosciences, Amherst, NH, USA) [6].

### 2.7. Statistical Methods

Descriptive data are expressed as percentages or as mean ± standard deviation (SD). A Student’s unpaired two-sided *t*-test was applied to continuous variables, while the chi-square test was used for analysing discrete variables. To gain more insight into individual changes in biomarker concentrations, repeated measures of variance were employed, as opposed to relying solely on group mean values. In the potential association between the concentration of CD5L with biomarkers of inflammation, a Pearson product-moment correlation analysis was performed. We used Kaplan–Meier analysis to examine CV mortality during the follow-up period. Multiple regression and ANCOVA analyses were used to evaluate the relation between CD5L concentration and several cofactors that potentially could influence the CD5L level. The covariates included in the multivariate and ANCOVA models were smoking, IHD, diabetes, Hb < 120 g/L, CRP, selenium conc. incl, EF < 40%, CD5L conc. incl, and active treatment. Cox proportional hazard regression analysis was used to evaluate the risk of CV mortality within 10 years. The independent variables included in the multivariate model were variables known to be associated with CV mortality: age, hypertension, diabetes, ischemic heart disease, obstructive pulmonary disease, Hb < 120 g/L, ejection fraction < 40% according to echocardiography, eGFR < 60 mL, 1st quartile of CD5L, TSH at inclusion, and 1st quartile of fT3.

*p*-values < 0.05 were considered significant, based on a two-sided evaluation. All data were analysed using standard software (Statistica v. 13.2, Dell Inc., Tulsa, OK, USA).

At both baseline and after 48 months, data on health-related quality of life (Hr-QoL) were gathered using the Short Form 36 (SF-36) questionnaire [25]. SF-36 is a widely used generic Hr-QoL tool consisting of 36 items, which are categorized into eight domains of Hr-QoL: physical functioning (PF), role limitations due to physical health issues (RP), bodily pain (BP), general health (GH), vitality (VT), social functioning (SF), role limitations due to emotional health issues (RE), and mental health (MH). These domains are then combined into two composite scores: the physical component score (PCS) and the mental component score (MCS). The PCS includes the domains PF, RP, BP, and GH, while the MCS encompasses VT, SF, RE, and MH (23). The scores are scaled from 0 to 100, with higher scores indicating better Hr-QoL.

## 3. Results

The baseline characteristics of the study population, which consisted of 359 individuals, are presented in Table 1. As shown, there were fairly equal proportions between the two genders, 72% had hypertension, 21% had ischaemic heart disease, and 22% presented with diabetes. Seven percent suffered from impaired systolic heart function, and the population had a documented selenium deficiency. The two populations (active vs. placebo) were well balanced (Table 1).

### 3.1. CD5L and Thyroid Hormones

**Figure 2:**
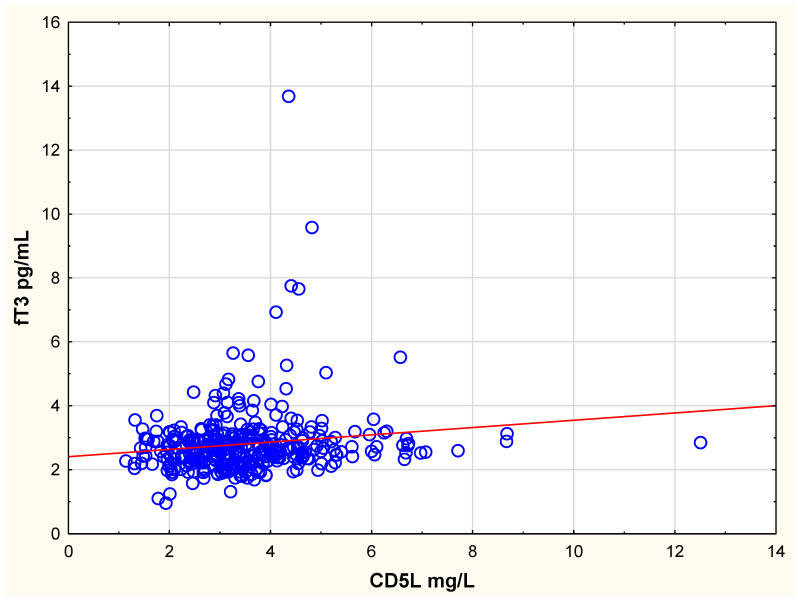
Scatterplot illustrating the relation between CD5L and fT3 in the study population at inclusion. Note: r = 0.14; *p* = 0.006; r^2^ = 0.021.

Addressing the hypothesis that circulating CD5L correlates with thyroid hormone concentrations, we examined the relation between CD5L concentration and the different thyroid hormones at baseline. We found significant positive correlations between CD5L and fT3 (r = 0.14; *p* = 0.006) (Figure 2) and fT4 (r = 0.11; *p* = 0.045), while an inverse relation was observed between CD5L and TSH (r = −0.11; *p* = 0.045).

However, the correlations were not overwhelmingly strong. There was no significant association between CD5L and hormonally inactive rT3 (r = 0.051; *p* = 0.34). In the multiple regression (Table 2), there were significant associations between CD5L and fT3 and TSH, respectively, but not with fT4. In the model, the variables “age” and “CRP” also had a significant impact on CD5L. The positive correlation between CD5L and fT3 was also present after 48 months (r = 0.22; *p* = 0.009).

### 3.2. CD5L and Inflammation

In the study population at inclusion, there were weak but significant and positive correlations between CD5L and the following biomarkers of inflammation: CRP (r = 0.17; *p* = 0.03), P-selectin (r = 0.10; *p* = 0.048), Galectin-3 (r = 0.20; *p* = 0.01), and ICAM-1 (r = 0.12; *p* = 0.03). To validate the relation between CD5L and CRP as an inflammatory biomarker, a multiple regression was performed including several thyroid hormones, and several covariates associated with increased cardiovascular risk. The analysis indicated a weak but significant association between CD5L and CRP (Table 2).

### 3.3. Effect of Intervention with Selenium and Coenzyme Q_10_ on CD5L Concentration

At inclusion in the project, there was no significant difference in the concentration of CD5L between the active and the placebo groups (active; 3.50 +/− 1.16 mg/L vs. placebo: 3.52 +/− 1.44 mg/L; *p* = 0.90). However, a significant but weak correlation between CD5L and serum concentration of selenium was found in the total population (t = 0.12; *p* = 0.03). After 48 months of supplementation, a significantly higher CD5L concentration was found in the active group, compared with the placebo group (active: 4.00 +/− 1.27 mg/L vs. placebo: 3.47 +/− 1.27 mg/L; *p* = 0.01). This increase was apparently due to a significantly elevated CD5L concentration in the active group (at inclusion: 3.50 +/− 1.16 mg/L vs. after 48 months: 4.00 +/− 1.27 mg/L; *p* = 0.002). In the placebo group, there was no significant difference in CD5L during the course of the study (at inclusion: 3.52 +/− 1.44 mg/L vs. after 48 months: 3.47 +/− 1.27 mg/L; *p* = 0.82).

**Figure 3:**
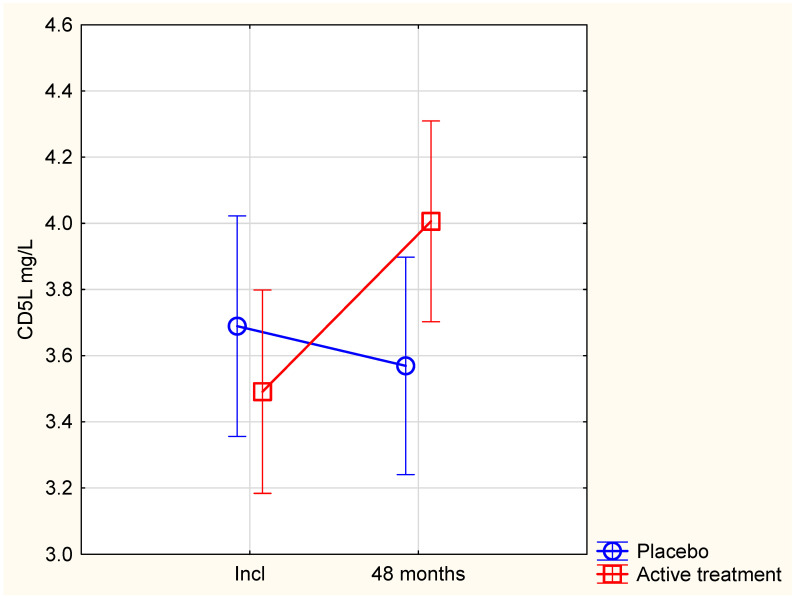
Repeated measure of variance showing level of CD5L in active versus placebo groups from inclusion to 48 months of treatment. Note: Current effect: F(1,135) = 10.5, *p* = 0.002. Vertical bars denote 0.95 confidence intervals.

When applying repeated measures of variance to better follow the individual changes, a significant difference in CD5L between the active and the placebo groups was apparent after 48 months (*p* = 0.002) (Figure 3).

To validate the changes, an ANCOVA analysis was performed including several cofactors that might influence the level of CD5L. From this evaluation, active treatment, Hb < 120 g/L and selenium level at inclusion, besides the level of CD5L at inclusion, were the only covariates that influenced the 48-month level of CD5L (Table 3).

As the effect of increasing CD5L concentrations could be a result of increased fT3 levels due to the supplementation, a correlation analysis between the delta values of fT3 and CD5L from inclusion to 48 months were performed. A significant correlation between delta fT3 and delta CD5L was observed (r = 0.23; *p* = 0.007), indicating that the effect of the intervention on CD5L is possibly in part a result of the increased fT3 level.

### 3.4. Relation Between CD5L and Health-Related Quality-of-Life and Effect of Supplementation with Selenium and Coenzyme Q_10_ on CD5L

**Figure 4:**
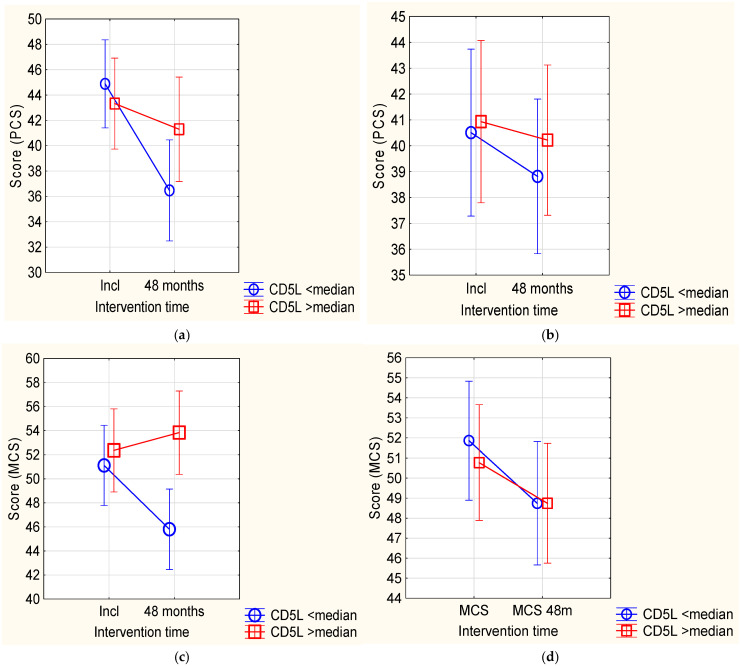
(**a**) Repeated measures of variance of the composite domain PCS from the SF-36 health-related quality-of-life questionnaire comparing CD5L at inclusion < median with CD5L > median in the placebo group. Note: Current effect: F(1,58) = 4.82; *p* = 0.032. Note: Vertical bars denote 0.95 confidence intervals. Note: PCS: Physical Component Score. (**b**) Repeated measures of variance of the composite domain PCS from the SF-36 health-related quality-of-life questionnaire comparing CD5L at inclusion < median with CD5L > median in the active group. Note: Current effect: F(1,68) = 0.17; *p* = 0.68. Note: Vertical bars denote 0.95 confidence intervals. Note: PCS: Physical Component Score. (**c**) Repeated measures of variance of the composite domain MCS from the SF-36 health-related quality-of-life questionnaire comparing CD5L at inclusion < median with CD5L > median in the placebo group. Note: Current effect: F(1,58) = 5.26; *p* = 0.025. Note: Vertical bars denote 0.95 confidence intervals. Note: MCS: Mental Component Score. (**d**) Repeated measures of variance of the composite domain MCS from the SF-36 health-related quality-of-life questionnaire comparing CD5L < median versus CD5L > median in the active group. Note: Current effect: F(1,68) = 0.17; *p* = 0.68. Note: Vertical bars denote 0.95 confidence intervals. Note: MCS: Mental Component Score.

To evaluate if CD5L was associated with Hr-QoL measures, we performed an analysis using the SF-36 questionnaire. From the eight domains that are covered by the instrument, significant differences between the placebo and the active treatment groups were detected in the scoring at 48 months for the two composite scores PCS and MCS that cover the physical and the mental domains, respectively (Figure 4a,c).

At inclusion in the placebo group, no difference in scoring between those having a CD5L concentration above versus below the median value was observed. However, after 48 months, those with a CD5L concentration below the median value displayed a significantly lower score in Hr-QoL compared with those with a CD5L concentration above the median. The latter participants reported on average a better Hr-QoL. In the other domains, we found no significant changes, probably due to the small sample size.

Analysing the active treatment group, however, no significant differences between the two groups (CD5L above vs. below median at inclusion) were noted after 48 months (Figure 4b,d).

### 3.5. Relation Between CD5L and Mortality Within 10 Years of Follow-Up

**Figure 5:**
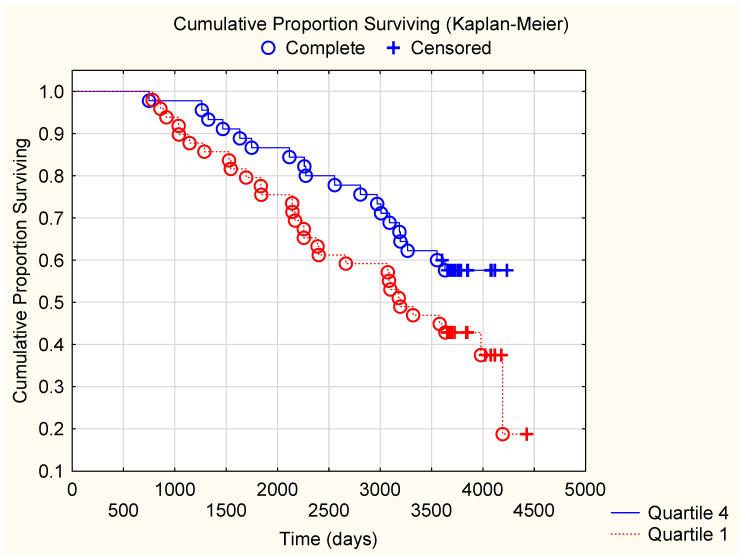
Kaplan–Meier graph illustrating survival from CV in the study population comparing those with CD5L concentration in the 1st quartile with those with CD5L concentration in the 4th quartile at inclusion during a follow-up period of 10 years. Note: Z = 2.34; *p* = 0.019. O: Complete; +: Censored.

Both CV- and all-cause mortality after 10 years was evaluated comparing the 1st (<2.68 mg/L) vs. 4th (>4.11 mg/L) quartiles of CD5L in both the placebo and in the active treatment groups. As shown in the Kaplan–Meier plot for the placebo group (Figure 5), a significantly higher CV mortality was found in the 1st quartile of CD5L compared to those in the 4th quartile (Q1: 26 deaths out of 49; 53.1% vs. Q4: 12 deaths out of 45: 26.7%; Chi2: 6.69; *p* = 0.009).

The corresponding results for all-cause mortality showed a borderline statistically significant difference (Q1; 30 deaths out of 49: 61.2% vs. Q4: 19 out of 45; 42.2%; Chi2: 3.39; *p* = 0.065). In the active treatment group, there were no significant differences in CV mortality or in all-cause mortality (CV mortality; Q1: 9 deaths out of 38 vs. Q4: 12 deaths out of 42; Chi2: 0.25; *p* = 0.62, all-cause mortality: Q1: 15 deaths out of 38 vs. Q4: 21 deaths out of 42; Chi2: 0.89; *p* = 0.34).

By applying a multivariate hazard regression analysis for CV mortality in the placebo group, we analysed the 1st quartile vs. the 4th quartile of CD5L concentration. Several well-known CV risk factors were included in the model, and a 10-year follow-up was applied (Table 4). The analysis indicated that those in the 1st quartile of CD5L concentration had a 2.2-fold increased CV mortality risk, even after being adjusted for several other risk factors including low fT3, hypertension, and diabetes.

## 4. Discussion

In this study, we tested the hypothesis that CD5L may serve as a biomarker reflecting selenium status and thyroid hormone activity by analysing serum samples from an intervention trial with selenium and CoQ_10_ in an elderly community-living population with baseline selenium deficiency. We hypothesised that the active supplementation would improve selenium-dependent deiodinase expression and local thyroid hormone activity leading to higher CD5L concentrations, with associations to improved thyroid hormone status, Hr-QoL, and reduced mortality.

The data obtained were in agreement with our assumptions. Firstly, we observed a positive relationship between fT3 and CD5L in a multiple regression analysis, which included several covariates that could potentially influence the CD5L levels. At inclusion, a significant positive correlation between serum selenium and CD5L concentrations was already present, in line with our recent research [18].

Secondly, supplementation with selenium and CoQ_10_ increased the level of CD5L as shown by using the repeated measures of variance approach (Figure 3) and in an adjusted model (Table 3). It is likely that the increase in CD5L, at least partly, is due to a local increase in fT3, as there was a significant correlation between the incremental increase in fT3 and the increase in CD5L upon supplementation. An inverse association between CD5L and TSH was also observed, in line with the negative feedback regulation of fT3 and TSH, and the initial description of CD5L as a positive biomarker of thyroid hormone activity [17]. Our results are thus fully compatible with increased peripheral deiodinase activity causing increased fT3 concentrations both locally and systemically upon selenium supplementation, as the deiodinases are selenoenzymes that are dependent on an adequate selenium supply. Accordingly, low fT3 concentrations were observed in subjects with genetic or autoimmune impairment of selenium transport and selenoprotein expression [26,27].

Our results concur with the results reported by Nock et al. showing that T3 stimulates CD5L production [17]. While Nock et al. based their findings on an animal model and an association observed in patients with hypo- or hyperthyroidism, we obtained the results from an active placebo-controlled randomized intervention study with a “healthy” community-living elderly population, implying that CD5L also provides information on selenium and thyroid hormone status on a population basis.

In our study, we also observed an association between CD5L and the inflammatory marker CRP at inclusion. However, in the ANCOVA evaluation in which the covariance with CD5L at 48 months was analysed, CRP did not reach significant impact. Due to the limited sample size, the number of covariates was restricted in order not to over-model. Yet an *inverse trend* between CD5L and CRP concentrations is compatible with their relationship to Hr-QoL and survival.

Of further interest, anaemia (Hb < 120 g/L), also significantly influenced the level of CD5L at 48 months as seen from the ANCOVA model. Anemia often has underlying conditions such as increased inflammation and oxidative stress, which partly may influence the level of CD5L [28,29]. Anaemia and hypoxia may also affect selenoprotein expression patterns [30], and inversely, low Se and the selenium transporter SELENOP status are associated with anaemia in CVD [31].

Our group has previously showed a relationship between Hr-QoL and the levels of thyroid hormones [6]. As, according to our hypothesis, we observed a close relationship between CD5L and the thyroid hormones, we also analysed the potential relationship between CD5L and Hr-QoL and found that CD5L levels were associated with the Hr-QoL score. Thus, in the placebo group, significant differences were noted in the two composite domains PCS and MCS at 48 months in those with a CD5L concentration below vs. above the median level at baseline. Interestingly, our results concur with the literature on patients with subclinical hypothyroidism [32], and it is tempting to speculate that both low CD5L and impaired Hr-QoL might be a result of low T3 activity. This notion agrees with a recent study indicating that T3, but not T4 or TSH, is significantly related to socio-economic well-being and longevity in general human physiology [33]. Notably, the baseline scores of the two domains of Hr-QoL correspond to the normative values from a Scandinavian general population at the corresponding age [34], and our population low in selenium had a steeper decrease in score with increasing age, compared with the normative population at the corresponding age. The reason for this is presently unclear. However, from the obtained results, we may suggest that low CD5L levels associate with symptoms related to impaired Hr-QoL not identified in the patient history that was taken for all patients before the inclusion in the main study project. Supplementation with selenium and coenzyme Q_10_ neutralised this difference, which supports our initial hypothesis on a health-relevant selenium-deficiency in our study groups.

Finally, and additionally, we observed an increased risk of cardiovascular mortality in the group with low CD5L concentrations at inclusion, not previously been reported. However, it is also important to note that the significant relationship between cardiovascular mortality and CD5L was only observed in the placebo group. The lack of a significance in the active treatment group may be due to the documented effects selenium and CoQ_10_ have on inflammation, oxidative stress and fibrosis, processes involved in ageing in the cardiovascular system, and thus also in the risk of cardiovascular mortality. By decreasing inflammation, oxidative stress, and fibrosing, it is plausible that this effect by the active treatment may have overshadowed the relation between CD5L and CV mortality and thus resulted in insignificant results.

A recent analysis on the potential health-supporting function of CD5L was conducted in an experimental model of sepsis indicating positive effects on survival with CD5L supplementation [35]. As CD5L has a multitude of functions, the explanation is uncertain. However, we previously found that impaired thyroid function was associated with increased CV mortality [6], which may explain the observed difference in CV mortality related to CD5L levels. This interpretation is supported by recent analysis of survival with breast cancer, where a positive association of selenium with deiodinase expression and hence an improved T3 activity and signalling was observed [36,37]. Yet, the proposed relevance of improved thyroid hormone metabolism in response to selenium supplementation as a central health- and longevity-supporting principle needs to be critically tested in sufficiently large prospective clinical trials. To this end, the herein reported characterization of CD5L as a survival-relevant biomarker constitutes an important prerequisite for monitoring such interventions.

## 5. Limitations

The sample size in this study was relatively small (n = 359), which may complicate the interpretation of the findings. However, given the highly significant difference between the two groups (active supplementation versus placebo), it is reasonable to infer that the observed effects are likely to reflect genuine biological changes. Despite this, our findings should be viewed as preliminary and hypothesis-generating, offering valuable insights that warrant further investigation.

The study was conducted in Sweden, a country where, like many European nations, the soil has a low selenium content and the population has insufficient dietary intake of this mineral. Similar low selenium levels are also found in countries like Australia and New Zealand. However, the positive outcomes of the intervention may not apply to regions or populations with adequate selenium levels and fully expressed deiodinases. The participants were not specifically selected for the study; instead, they were chosen because they resided in the same rural community and were within the same age range. This could introduce some bias, as individuals with known or unknown health issues, poorer well-being, or those seeking a diagnosis or treatment modification might have been more inclined to participate. Nevertheless, since the participants were randomly assigned to either the active treatment or placebo group, we expect that the health status of those receiving the active treatment was similar to those receiving the placebo, as evidenced by the balanced baseline characteristics.

Moreover, the study population represented a specific age group of the elderly, which limits the ability to generalize the findings to younger age groups. Finally, as the study sample was ethnically homogeneous, consisting of a Caucasian population, it remains unclear whether the findings are applicable to other ethnic groups.

## 6. Conclusions

Our data suggest that CD5L may be suitable as a new and additional biomarker for improved thyroid function, adequate selenium status, and consequently reduced mortality risk. Dietary supplementation of selenium and CoQ_10_ for four years significantly increased the level of CD5L and improved Hr-QoL and survival odds. In the placebo group, low levels of CD5L were associated with significantly higher CV mortality within 10 years, while mortality was reduced upon supplementation. Whether the association between CD5L, selenium, Hr-QoL, and CV mortality reflects an improved thyroid hormone metabolism secondary to the intervention is a possible explanation, but this conclusion is currently uncertain and should be investigated further.

## Figures and Tables

**Table 1:** Baseline characteristics of the study population at inclusion, stratified according to active supplementation of selenium and CoQ_10_ and placebo.

	Active Treatment n = 179	Placebo n = 180	*p*-Value
Age years, mean (SD)	77 (3.6)	77 (3.4)	
Sex, Males/Females, n	96/83	95/85	
**History**			
Smoking, n (%)	16 (8.9)	11 (6.1)	0.31
Hypertension, n (%)	127 (70.9)	133 (73.9)	0.53
IHD, n (%)	35 (19.6)	39 (21.7)	0.62
Diabetes, n (%)	40 (22.3)	38 (21.1)	0.78
NYHA class I, n (%)	97 (54.2)	88 (48.9)	0.32
NYHA class II, n (%)	49 (27.4)	51 (28.3)	0.84
NYHA class III, n (%)	32 (17.9)	38 (21.1)	0.44
NYHA class IV, n (%)	0	0	
Unclassified, n (%)	1 (0.5)	3 (1.7)	
**Medications**			
Beta blockers, n (%)	62 (34.6)	56 (31.1)	0.48
ACEI/ARB, n (%)	39 (21.8)	52 (28.9)	0.12
Diuretics, n (%)	55 (30.7)	71 (39.4)	0.08
**Examinations**			
EF < 40%, n (%)	12 (6.7)	14 (7.8)	0.69
s-selenium pre-intervention µg/L, mean (SD)	66.7 (16.0)	66.3 (17.9)	0.80

Note: ACEI: Angiotensin-converting enzyme-inhibitors; ARB: Angiotensin receptor blockers; EF: Ejection fraction; IHD: Ischemic heart disease, NYHA: New York Heart Association functional class; SD: Standard Deviation. Note: Values are means ± SDs or frequency (percent). Note: Student’s unpaired two-sided *t*-test was used for continuous variables, and the chi-square test was used for analysis of one discrete variable.

**Table 2:** Multiple regressions using CD5L at inclusion as dependent variable of the study population.

Variable	β	Standard Error of β	B	Standard Error of B	T-Value	*p*-Value
**Intercept**			−1.76	2.41	−0.73	0.47
**Age**	0.19	0.08	0.07	0.03	2.34	0.02
**Diabetes**	−0.02	0.08	−0.05	0.23	−0.20	0.84
**NYHA 3**	−0.10	0.08	−0.35	0.27	−1.29	0.20
**Smoking**	−0.15	0.08	−0.93	0.48	−1.91	0.06
**CRP**	0.15	0.08	0.01	0.006	1.88	0.045
**Hb < 120 g/L**	−0.10	0.08	−0.39	0.30	−1.27	0.21
**fT3**	0.22	0.09	0.24	0.10	2.46	0.01
**TSH**	−0.17	0.08	−0.23	0.11	−2.12	0.04
**fT4**	−0.05	0.08	−0.02	0.03	−0.58	0.56
**Selenium, µg/L, incl**	−0.05	0.08	−0.004	0.006	−0.60	0.55

Note: r = 0.39; r^2^ = 0.15; F(10,147) = 2.68; *p* = 0.005. Note: CRP: C-reactive protein, NYHA: New York Heart Association functional class.

**Table 3:** Analysis of covariance using CD5L after 48 months as dependent variable.

Effects	Sum of Squares	Degrees of Freedom	Mean Squares	F	*p*
**Intercept**	10.39	1	10.39	10.27	0.002
**Smoking**	2.77	1	2.77	2.74	0.10
**IHD**	0.22	1	0.22	0.22	0.64
**Diabetes**	0.02	1	0.02	0.02	0.90
**Hb < 120 g/L**	6.70	1	6.70	6.63	0.01
**CRP**	3.1	1	3.1	3.1	0.09
**Selenium µg/L, incl.**	4.22	1	4.22	4.17	0.047
**EF < 40%**	0.90	1	0.90	0.90	0.35
**CD5L mg/L incl.**	50.15	1	50.15	49.61	<0.0001
**Active treatment**	6.54	1	6.54	6.47	0.014
**Error**	48.52	48	1.01		

Note: CRP: C-reactive protein; EF: Ejection fraction; IHD: Ischemic heart disease; incl.: at inclusion.

**Table 4:** Cox proportional hazard regression analysis evaluating the risk of cardiovascular mortality: CD5L in 1st quartile vs. 4th quartile was evaluated in the placebo group at inclusion, including several well-known risk factors for mortality in a multivariate model after 10 years of follow-up after 4 years of intervention.

Variables	Hazard Ratio	95%CI	*p*-Value
**Age**	1.10	1.0–1.20	0.05
**Hypertension**	2.79	1.21–6.46	0.02
**Diabetes**	2.67	1.21–5.89	0.014
**IHD**	6.15	2.66–14.20	<0.0001
**Obstr pulm dis**	0.44	0.14–1.32	0.14
**Hb < 120 g/L**	0.85	0.29–2.46	0.77
**EF < 40%**	0.73	0.23–2.32	0.59
**eGFR < 60 mL**	1.73	0.52–5.72	0.37
**CD5L Q1 < 2.68 mg/L**	2.24	1.02–4.91	0.04
**TSH, ulU/mL**	0.67	0.44–1.02	0.06
**fT3 Q1 < 2.28 pg/mL**	1.33	0.53–3.32	0.54

Notes: EF: Ejection fraction; eGFR: estimated glomerular filtration rate; fT3: free part of thyroxine; IHD: Ischemic heart disease; Obstr pulm dis: obstructive pulmonary disease; Q1: 1st quartile; TSH: thyroid stimulating hormone.

## Data Availability

Under Swedish Law, the authors cannot share the data used in this study and cannot conduct any further research other than what is specified in the ethical permissions application. For inquiries about the data, researchers should first contact the owner of the database, the University of Linköping. Please contact the corresponding author with requests for and assistance with data. If the university approves the request, researchers can submit an application to the Regional Ethical Review Board for the specific research question that the researcher wants to examine.
